# Host–Guest Polymer Complexes

**DOI:** 10.3390/polym10080911

**Published:** 2018-08-13

**Authors:** Alan E. Tonelli, Ganesh Narayanan, Alper Gurarslan

**Affiliations:** 1 Fiber & Polymer Science Program College of Textiles, North Carolina State University, Campus Box 8301, 2401 Research Drive, Raleigh, NC 27695-8301, USA; gnaraya@ncsu.edu; 2 Faculty of Textile Technologies and Design, Istanbul Technical University, Inonu Cad. No 65 Gumussuyu, Beyoglu, Istanbul 34437, Turkey; gurarslan@itu.edu.tr

The discovery of host–guest complexation has resulted in significant advancements in various facets of materials science, including: drug delivery, tissue engineering, etc. Various host molecules such as cyclodextrins (CD), urea, thiourea, cyclo-triphosphazines, pillar[6]arene, cucurbit[6]uril, and calix[4]arene have been identified as undergoing inclusion complexation with a range of guest molecules. Among these host molecules, cyclodextrins are well-known for their complexation capabilities with a variety of guest molecules. Through such complexation, the stability of the guest against thermo-chemical, environmental, light, and air degradation is ensured. In addition, the widespread availability of CD-derivatives (randomly methylated, 2-hydroxypropylated, etc.) has partly contributed to their widespread use in applications extending from textiles to pharmacologics to nuclear waste removal. Another benefit of forming such complexes is that they improve the polymeric properties (for example, mechanical, rheological, and thermal properties) by coalescing the guest polymers from their CD inclusion complexes.

While the host–guest complexation occurs predominantly via non-covalent interactions, the underlying mechanism differs among the studied guest molecules, with predominant complexation occurring via hydrogen and ionic bonding, van der Waals and hydrophobic forces/interactions. Even though the mechanisms differ between the chosen host/guest molecules, a common criterion for successful complexation remains lowered Gibbs free energy, which can occur either by an enthalpy- or an entropy-driven process. The thermodynamics of the host–guest complexation and associated binding constants are typically characterized using spectroscopy-based techniques such as nuclear magnetic resonance, UV-vis, mass spectrometry, fluorescence-based spectroscopy or calorimetric techniques such as differential scanning and isothermal titration calorimetry [1]. With such a wide range of possibilities in terms of guest and host molecules in host–guest chemistry, the purpose of this special Issue is to provide the latest advancements as well as novel applications of these host–guest compounds.

This Special Issue consists of 15 articles, including four comprehensive review articles, written by experts in the field. The first eleven articles are dedicated to the synthesis and characterization of host–guest containing compounds for the fabrication of devices, in particular for biomedical applications. The next four articles are comprehensive review articles discussing the state-of-art synthesis, characterization, and fabrication of various host–guest complexes, including CD–polymer, amylose–polymer, polysaccharide–metal ion, and urea–polymer inclusion complexes. In this series, the first research article reports the synthesis of novel host–guest supramolecular hydrogels comprised of pNIPAm microgels bearing poly[acrylic acid] with attached β–CDs as the host and adamantane–dextrans as the guest molecules. By forming in situ complexation, hydrogels were synthesized that showed sol–gel transitions at physiologically relevant temperatures (37 to 41 °C) [2].

One of the advantages of such a system is in the field of the controlled release/retention of small molecules, mediated in part by the concerned host and the guest molecules. Likewise, the second study reports the fabrication of host–guest aerogels containing fluorescently active quantum dots. In the same way that the interaction with CD protects the small molecules from degradation, this study demonstrated the fluorescence-retention potential of quantum dots upon complexation/interaction with β–CD [3]. 

A major challenge in tissue engineering remains the lack of oxygen and frequent bacterial infection during tissue regeneration. By complexation, the third and fourth articles in this Special Issue address these two major challenges in tissue regeneration [4,5]. By encapsulating oxygen into the cavities of α–CD and α–CD polymers, in vitro cell studies demonstrated a marked reduction in hypoxia–reoxygenation injury, leading to favorable cell growth and lowered cell mortality [4]. Similar to the approach reported by the first two studies [2,3], the fourth study reported the fabrication of a polyrotaxane (PR) containing α–CD and poly [ethylene glycol] containing silver sulfadiazine. When encapsulated in the PR matrix, silver sulfadiazine demonstrated not only superior antibacterial activity against *E. coli* and *S. aureus*, but also resistance to light-induced degradation [5]. The fifth article reports a novel technique to impart anti-bacterial activity by surface grafting CD containing triclosan to a woven polypropylene mesh, resulting in the sustained release of triclosan, making this medical device suitable in clinical settings [6].

The next three articles reported the synthesis and electrostatic-force-induced complexation of anionic polyelectrolytes and dendrigraft poly(l-Lysine) [7], poly (γ-glutamic acid) and ethyl lauryl alginate [8], pillar[6]arene containing polyelectrolytes [9]. As shown by these studies, one of the advantages of utilizing polyelectrolytes is in the utilization of a layer-by-layer approach for the fabrication of thin films, which could be subsequently formulated for various applications, including: antibacterial films/patches.

In the past two decades, our group (https://textiles.ncsu.edu/blog/team/alan-tonelli/) has reported a facile technique to render incompatible polymers more compatible by threading through the CD cavity and subsequently removing the CD, resulting in improved compatibility between otherwise incompatible blends. In addition, over the years, we have extended this technique to urea–polymer inclusion complexes as well. Similar to this established approach, Liu et al. report an improved ability to crystallize and compatibility between PLLA/PDLA by coalescing from a common inclusion complex [10]. In a similar vein, but without using host molecules for complexation, the next article discusses directional alignment of polyfluorene copolymers at patterned solid-liquid interfaces [11]. The final research article in this Special Issue reports a novel complexation between the collagen (host) and polyphosphates (guest). This is particularly crucial because collagen forms the basic structural unit of human anatomy and polyphosphates are superior metabolic fuel for the synthesis and maintenance of extracellular tissue [12].

The next section of this Special Issue contains review articles from expert researchers on the synthesis, characterization and fabrication of state-of-the-art devices based on inclusion complexation. The first review, from our group, is based on the fabrication of aliphatic polyester nanofibers containing uncomplexed and complexed CDs or inclusion complexes. In this review, we report various possibilities for improving the topographical, mechanical and cell-adhesive properties of aliphatic polyester nanofibers via an electrospinning process [13]. The next two articles discuss the synthesis and characterization of novel inclusion complexes between amylose and polymers, and polysaccharides and metal ions [14,15]. Unlike most of the articles reported in this Special Issue, which are based on CDs, complexation with polysaccharides is unique and hard to accomplish; this review provides a useful starting resource for further research. The final review article, also from our group, discusses the complexation between host (CD and urea) and guest polymeric molecules. This review discusses the morphological and thermal changes observed in the homo- and co-polymer guests upon coalescence, as well as changes in their crystallizability [16].

Overall, we anticipate this Special Issue to be a ready reference for those interested in the synthesis, characterization and fabrication of inclusion complexes between guest polymers and host molecules. We hope these reviews will encourage advanced research on host–guest inclusion complexes. As the editors of this Special Issue, we acknowledge and offer our sincere thanks to the authors and the reviewers for their invaluable contribution to this Special issue.
